# Comparison of Thulium Fiber Laser versus Holmium laser in ureteroscopic lithotripsy: a Meta-analysis and systematic review

**DOI:** 10.1186/s12894-024-01419-6

**Published:** 2024-02-19

**Authors:** Xiaoyu Tang, Shaojie Wu, Zhilong Li, Du Wang, Cheng Lei, Tongzu Liu, Xinghuan Wang, Sheng Li

**Affiliations:** 1 Department of Urology, Zhongnan Hospital of Wuhan University, 169 Donghu Road, Wuhan, 430071 China; 2 Department of Biological Repositories, Cancer Precision Diagnosis and Treatment and Translational Medicine Hubei Engineering Research Center, Zhongnan Hospital of Wuhan University, Wuhan, 430071 China; 3 The Institute of Technological Sciences, Wuhan University, Wuhan, 430072 China

**Keywords:** Thulium fiber laser, Ho:YAG, Urolithiasis, Ureteroscopic lithotripsy, Systematic review, Meta-analysis

## Abstract

**Objectives:**

To compare the efficacy and safety of thulium fiber laser (TFL) to holmium: YAG (Ho: YAG) laser in ureteroscopic lithotripsy for urolithiasis.

**Methods:**

PubMed, Web of Science, Embase, CENTRAL, SinoMed, CNKI database, VIP and Wanfang Database were systematically searched for all relevant clinical trials until September 2023. References were explored to identify the relevant articles. Meta-analysis was carried out for the retrieved studies using RevMan5.4.1 software, and the risk ratio, mean difference and 95% confidence interval were expressed. Statistical significance was set at *p* < 0.05. The main outcomes of this meta-analysis were stone-free rate (SFR), perioperative outcomes and intraoperative or postoperative complications.

**Results:**

Thirteen studies, including 1394 patients, were included. According to the results of pooled analysis, TFL was associated with significantly higher stone-free rate (SFR) [0.52, 95% CI (0.32, 0.85), *P* = 0.009], shorter operation time [-5.47, 95% CI (-8.86, -2.08), *P* = 0.002], and less stone migration [0.17, 95% CI (0.06, 0.50), *P* = 0.001]. However, there was no significant difference in terms of the laser time, duration of hospital stay, drop of hemoglobin level, total energy, postoperative ureteral stenting, the incidence of intraoperative complications or postoperative complications between TFL and Ho: YAGs.

**Conclusion:**

The findings of this study demonstrated several advantages of TFL in terms of higher SFR, shorter operative time and less stone migration.

**Trial registration:**

The protocol of this systematic review was listed in PROSPERO (www.crd.york.ac.uk/PROSPERO) (Protocol number: CRD42022362550).

## Background

Since the first clinical application of the holmium: yttrium aluminum garnet (Ho: YAG) laser in 1992, it has quickly come to dominate the field of endourological lithotripsy. It has been considered the gold standard for ureteroscopic lithotripsy due to its clinical efficacy, safety, and durability [1]. However, this laser lithotripsy still has some drawbacks, including poor endoscopic vision, stone retropulsion, large machine size [2], low energy efficiency, high tissue thermal damage, fewer options for lithotripsy parameters, and relatively thick fiber diameter [3, 4].

Thulium fiber laser (TFL) lithotripsy is one of the latest laser therapy techniques. Since its first clinical application in 2018 [5], people have been paying increasing attention to it. Preclinical studies have shown that TFL has a higher water absorption coefficient, smaller optical penetration depth, and lower stone ablation threshold compared with Ho: YAG [6, 7]. In addition, TFL can transmit high-power laser beams more efficiently through smaller, more flexible optical fibers, which makes their devices more portable, more conducive to the dusting of stones, and reduces stone retropulsion [2].

In the last 2–3 years, an increasing number of studies have analyzed its clinical application for urolithiasis management [5, 8–10], and it may challenge Ho: YAG as the laser of choice due to various advantageous properties; it may even mark a turning point in endourology.

However, due to differences in the results of different studies, whether TFL is superior to Ho: YAG in patients with urolithiasis is unclear, and in-depth evaluation of this issue has important clinical implications. Therefore, we conducted a systematic review and meta-analysis, hoping to provide accurate evidence for the clinical use of laser lithotripsy.

## Materials and methods

This systematic review was based on the recommendations of the Cochrane Collaboration for Systematic Reviews and reported in accordance with the PRISMA statement [11]. The protocol of this systematic review was listed in PROSPERO (www.crd.york.ac.uk/PROSPERO) (Protocol number: CRD42022362550) [12].

### Eligibility criteria

According to PICOS [11], the following criteria were considered:

- Participants: Patients who were clinically diagnosed with urolithiasis and required ureteroscopic lithotripsy were included without limitations of race or nationality. However, patients with urinary infections, congenital anatomic abnormalities, urothelial tumor(s), and no stones when diagnosed with the ureteroscope were excluded.
- Interventions: TFL lithotripsy.
- Comparator: Ho: YAG lithotripsy.
- Outcomes: The primary endpoint: Stone-free rate (SFR); The SFR was evaluated using CT or ultrasound at one or three months of postoperative follow-up. The two different SFR definitions are zero residual fragments and no residual fragments more than 3 mm. The secondary endpoint: (a) Perioperative outcomes: operation time (min), laser time (min), hospital stay (day), drop in hemoglobin(g/dL), total energy (KJ), postoperative ureteral stenting rate; (b) Intraoperative and postoperative complications.
- Study design: Randomized controlled trial (RCT), non-RCT.

The following studies were excluded: (a) case reports, editorials, comments, letters, and in vitro studies; (b) studies that lacked of main data, with obvious errors or inconsistent data that received no response after contacting the authors of these studies; and (c) when two studies came from the same institution with the same results, the first author of the study was contacted to clarify the differences. If both studies were derived from the same experimental results, the study with better quality or more comprehensive information was selected.

### Information sources and search strategy

The PubMed, Web of Science, Embase, the Cochrane Central Register of Controlled Trials (CENTRAL), SinoMed (China Biology Medicine disc), Chinese National Knowledge Infrastructure (CNKI) databases, VIP (China Science and Technology Journal Database) and Wanfang database were searched for all relevant clinical trials until September 2023. No regional, publication status, temporal, or language restrictions were set. The related reviews and the references of each included study were manually searched to ensure that no potentially eligible studies were missed. Different searches were carried out using the following terms and keywords: “thulium”, “fiber”, “laser”, “TFL”, “holmium”, “Ho: YAG”, “lithotripsy”, “urolithiasis”, “stone”, and “calculi”. Two evaluators (TXY and WSJ) carried out the search and cross-checked the results independently.

### Data extraction

Two researchers (TXY and WSJ) independently screened the studies and read the full text to determine the final studies to be included and then extracted content from the included studies. The criteria were as follows: (a) basic information of the included research, such as the title of the study, the name of the first author, and the year of publication; (b) baseline characteristics of the patients, including the inclusion criteria, sample size, the age, gender, and stone size; (c) methods and processes of research design, including the process of sampling and grouping, specific details of intervention measures, follow-up time, lost follow-up rate, and reasons for loss; and (d) outcome indicators and measurement data. In the studies with multiple experimental and control groups, only the experimental and control groups related to this text or preferentially selected for matching analysis were extracted. Some research data reporting only the medians, quartiles, or extreme value ranges of the continuous variable were converted to means and standard deviations (SDs) according to the formula [13, 14].

### Methodological quality assessment

For RCTs, two researchers (LZL and WD) independently assessed the methodological quality of studies in accordance with the Jadad scale [15]. The following three aspects were evaluated: (1) the randomization method and its concealment, (2) the blind method, and (3) the number of cases that dropped out and were lost to follow-up with their reasons. The total score on the Jadad scale was 5 points, and a score ≥ 3 points was considered high quality.

For non-RCTs, including cohort or case‒control studies, the researchers (LZL and WD) assessed the methodological quality based on the modified Newcastle‒Ottawa Scale (NOS) [16]. The following three aspects were evaluated: (1) study population selection, (2) comparability between intervention and control groups, and (3) outcome assessment [16]. The total available NOS score was 9 points, and a score > 5 points was considered high quality.

### Data analysis

The RevMan 5.4.1 software provided by the Cochrane Collaboration was used for data analysis. We calculated pooled risk ratios (RRs) for dichotomous variables, weighted mean differences (WMDs) for continuous variables, and their 95% confidence intervals (CIs). In addition, before data analysis, the chi-square test and I^2^ test [17] were used to evaluate the heterogeneity of the included studies. If *P* value < 0.10 or I^2^ > 50%, it was defined as statistical heterogeneity [18]. And the random effects model was adopted for data analysis after the source of heterogeneity was analyzed and the influence of obvious clinical heterogeneity was excluded [19]. If not, the fixed effects model was used, indicating acceptable heterogeneity [20]. Significant clinical heterogeneity was processed by sensitivity analysis, subgroup analysis, or only descriptive analysis. A value of *p* < 0.05 was considered statistically significant.

We conducted sensitivity analyses to investigate the impacts of individual studies and examine the robustness of the primary results by removing each study sequentially. Furthermore, subgroup analyses were performed based on stone size, stone location, ureteroscope, study design, follow-up time, MOSES technique, dusting technique, stone density and laser setting to explore the sources of heterogeneity and assess the impacts of the overall estimates.

## Results

### Search results

**Fig. 1:**
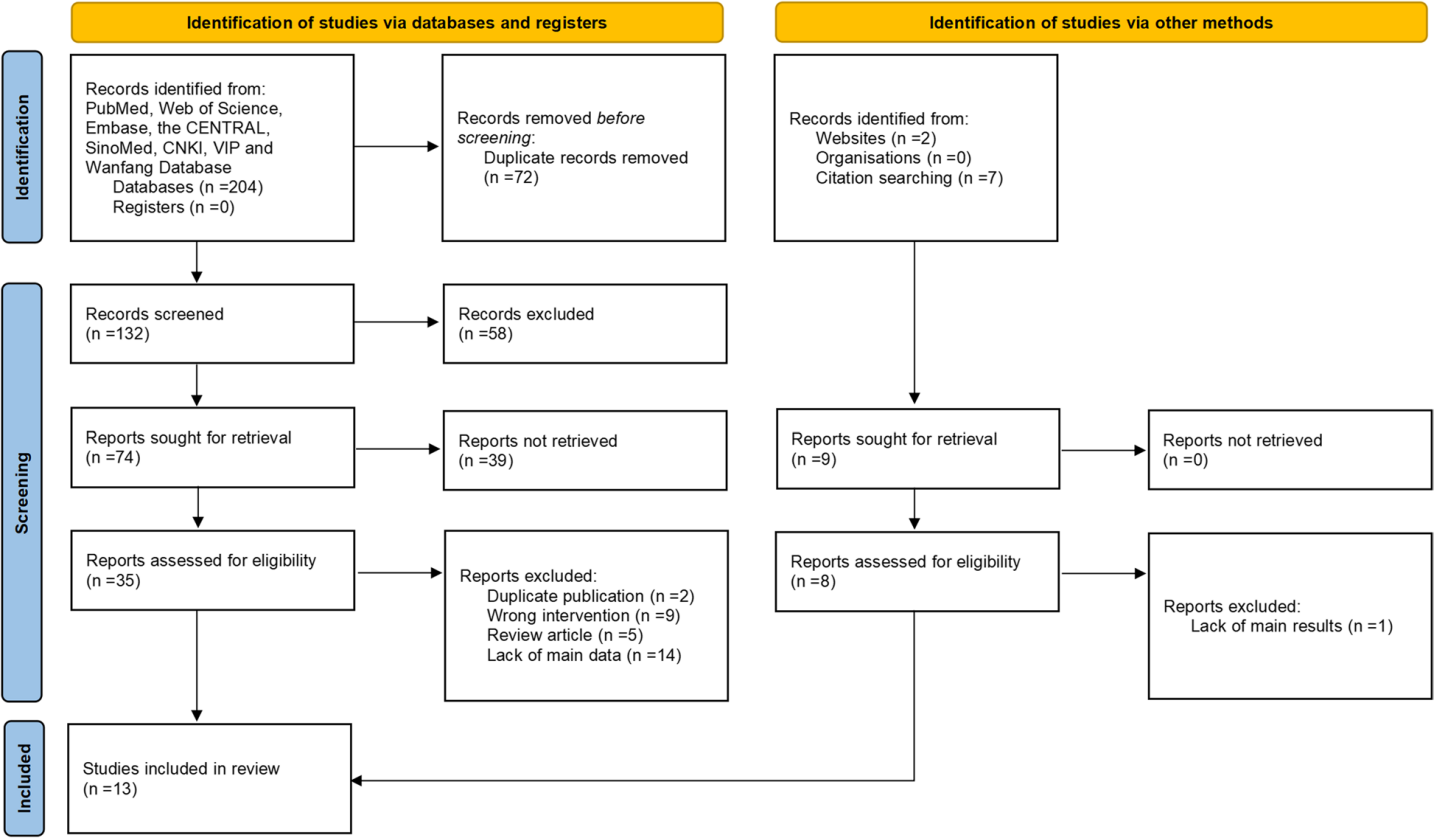
The screening process in the meta-analysis

An initial search found 213 related publications, including 9 supplements from another source. Among these studies, 72 were excluded due to duplicate records, and 58 were excluded after reading the title and abstract. The full-text data of 43 articles were reviewed. Furthermore, 30 articles were excluded because they did not meet the inclusion criteria. The final 13 studies [21–33], including 1394 patients, were included in this meta-analysis (Fig. 1).

### Study characteristics and quality

All the studies were published between 2020 and 2023. There were six randomized controlled trials and seven cohort studies. These studies were conducted in different countries, including China, India, the United States, Norway, Romania, France and Russia. The baseline characteristics were comparable between the TFL and Ho: YAG groups, with no statistically significant differences in sex, age, location (left/ right), stone diameter, stone volume, stone density (Table 1). Most studies evaluated the presence of residual stones by KUB or CT after 1 or 3 months. Bogdan [28] performed a second-look flexible ureteroscopy 3 months later to evaluate for residual stones. The median Jadad score for RCTs was 2.5 (range 1–5). Only one study [26] explicitly mentioned blinding for patients and researchers, which was acceptable because it was difficult for medical staff to be blinded to the type of laser used (instruments and parameters vary for different lasers). The median NOS score for non-RCTs was 7 (range 5–9), indicating the high quality of these studies (Table 1).

**Table 1:** Baseline characteristics of patients in the meta-analysis

Study	Study design	Country	Location	Ureteroscope	Treatment regimen	No. of patients	Male/ female	Age (mean ± SD, years)	Left/ Right	Mean diameter (mean ± SD, mm)	Mean Volume (mean ± SD, mm^3^)	Stone density (mean ± SD, HU)	Laser setting	MOSES technique	Dusting technique	Follow-up time (months)	NOS score	Jadad score
Huo YF 2020 [27]	non-RCT	China	Ureter	rigid ureteroscope	TFL	56	40/16	42.6±4.1	30/26	11±5	NA	NA	NA	N	N	12	7	NA
					Ho: YAG	46	35/11	41.3 ± 4.5	25/21	12 ± 4	NA	NA	NA					
Martov A G 2020 [23]	RCT	India	Ureter	semi-rigid ureteroscope	TFL	87	50/37	48.1 ± 5.2	45/42	12.2 ± 0.1	NA	1001 ± 266	1 J, 10 Hz	N	N	1	NA	2
					Ho: YAG	87	48/39	46.4 ± 4.3	43/44	11.3 ± 0.1	NA	994 ± 214	1 J, 10 Hz					
Popov S V 2020 [33]	non-RCT	Russia	Ureter	semi-rigid ureteroscope	TFL	60	24/26	51 ± 8	NA	8.1 ± 1.8	NA	1231 ± 192	0.8 J, 10 Hz	N	N	NA	7	NA
					Ho: YAG	50	30/30	51 ± 8	NA	8.3 ± 1.5	NA		0.8 J, 10 Hz					
Ghazi A 2021 [31]	non-RCT	USA	Kidney	NA	TFL	31	NA	56.13 ± 13.32	NA	NA	1150.8 ± 2254.8	1045.1 ± 109.19	0.4 J, 60 Hz	N	Y	1	6	NA
					Ho: YAG	31	NA	54.85 ± 12.09	NA	NA	1088.9 ± 1612.9	803.25 ± 302.6	0.4 J, 60 Hz					
Azilgareeva C 2022 [21]	RCT	Russia	Kidney/Ureter	flexible ureteroscope	TFL	29	NA	52 ± 14.1	NA	11.5 ± 3.3	322.0 ± 249.4^a^	1163.0 ± 367.1	NA	N	N	3	NA	2
					Ho: YAG	25	NA	55.4 ± 14.3	NA	12.4 ± 4.1	508.9 ± 248.2^a^	1124.0 ± 329.0	NA					
Bogdan G 2022 [28]	non-RCT	Romania	Kidney	flexible ureteroscope	TFL	59	32/27	48.94 ± 15.93	18/41	13.25 ± 4.74	NA	1045.1 ± 109.19	0.5 J, 30 Hz	Y	Y	3	9	NA
					Ho: YAG	187	105/82	47.51 ± 14.72	79/108	13.05 ± 3.27	NA	1020.45 ± 110.85	0.4 J, 80 Hz					
James R 2022 [24]	non-RCT	USA	Kidney/Ureter	NA	TFL	51	25/26	NA	NA	8.28 ± 3.75	NA	NA	NA	N	N	NA	7	NA
					Ho: YAG	51	31/20	NA	NA	8.56 ± 2.29	NA	NA	NA					
Singh A 2022 [25]	RCT	India	Kidney/Ureter	NA	TFL	30	NA	46.63 ± 13.73	11/18	NA	601.65 ± 673.08	1043.07 ± 290.4	0.05-1 J, 50-300HZ	N	N	1	NA	1
					Ho: YAG	30	NA	50.31 ± 13.24	19/11	NA	247.91 ± 227.01	1013.23 ± 328.1	0.2-2 J, 10-40HZ					
Oyvind U 2022 [26]	RCT	Norway	Kidney/Ureter	flexible/semi-rigid ureteroscope	TFL	60	38/22	53 ± 22.24	37/23	11.4 ± 6.12^a^	NA	896 ± 486.7^a^	0.4 J, 6 Hz	N	Y	3	NA	5
					Ho: YAG	60	39/21	55.18 ± 14.23	34/26	12.9 ± 8.38^a^	NA	911 ± 429.6^a^	0.4 J, 6 Hz					
Ankit G 2023 [22]	RCT	India	Ureter	semi-rigid ureteroscope	TFL	40	25/15	44.93 ± 14.11	NA	NA	282.45 ± 139.79	1135.3 ± 317.04	0.8-1 J, 10-12HZ	N	N	1	NA	3
					Ho: YAG	40	32/8	47.72 ± 12.88	NA	NA	279.49 ± 312.52	1131.75 ± 283.03	0.8-1 J, 10-12HZ					
Bertrand D 2023 [32]	non-RCT	France	Kidney/Ureter	flexible/rigid ureteroscope	TFL	100	43/57	60.1 ± 17.7	NA	14.6 ± 8.8	NA	NA	NA	N	N	3	7	NA
					Ho: YAG	76	43/33	57 ± 18.2	NA	11.6 ± 5.6	NA	NA	NA					
Castellani D 2023 [30]	non-RCT	Global	kidney	flexible ureteroscope	TFL	284	183/101	53.15 ± 15.04	NA	12.31 ± 5.02	NA	1146.67 ± 261.59	NA	N	Y	3	9	NA
					Ho: YAG	284	183/101	56.29 ± 14.80	NA	13.14 ± 5.87	NA	1141.91 ± 425.89	NA					
Christopher R H 2023 [29]	RCT	USA	Kidney/Ureter	flexible/semi-rigid ureteroscope	TFL	56	26/30	59 ± 12.6^a^	NA	8.9 ± 3.8^a^	288 ± 217.8^a^	NA	0.8 J, 8 Hz	Y	N	2	NA	3
					Ho: YAG	52	31/21	61 ± 11.1^a^	NA	8.4 ± 4.4^a^	319 ± 342.2^a^	NA	0.8 J, 8 Hz					

*RCT* randomized controlled trial, *SD* standard deviation, *NA* not available, *NR* not reported, *NOS* Newcastle–Ottawa score, *Y* yes, *N* no

^a^mean and standard deviation were converted from median and quartile of reported continuous variables

### Stone-free rate

**Fig. 2:**
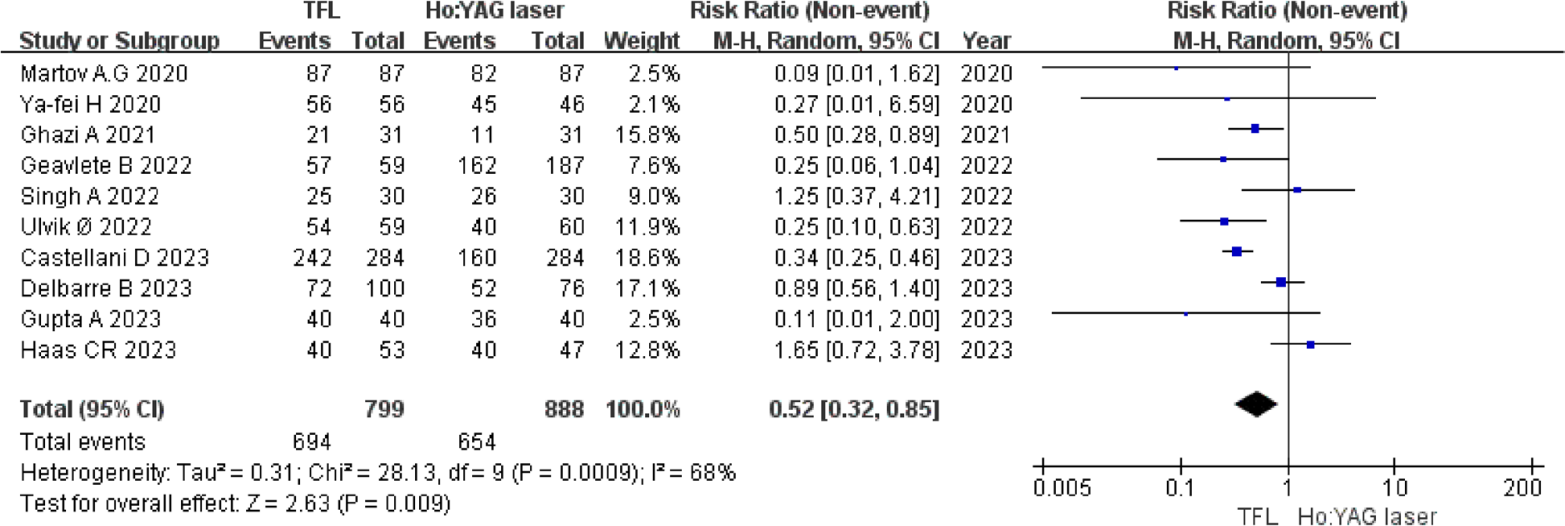
Forest plot comparing TFL and Ho: YAG in terms of stone-free rate

Ten studies [22, 23, 25–32] reported the SFR. The SFRs of the TFL and Ho: YAG groups were 86.9% and 73.6%, respectively. The heterogeneity was acceptable among the studies (*P* = 0.0009, I^2^ = 68%). The findings revealed that the TFL was associated with a higher SFR than Ho: YAG, and the difference between the two groups was statistically significant [0.52, 95% CI (0.32, 0.85), *P* = 0.009] (Fig. 2).

### Perioperative outcomes

For the perioperative outcomes, 11 studies [21–28, 30, 32, 33] reported data on operation time, 8 studies [21–23, 26, 29–31, 33] reported laser time data, 4 studies [21–23, 25] reported data on duration of hospital stay, 3 studies [21, 22, 25] reported the drop in hemoglobin, 5 studies [21, 22, 26, 29, 31] reported the total energy used in the lithotripsy process, and the remaining 3 studies [23, 26, 32] reported postoperative ureteral stenting rates. The findings suggested that the operation time of the TFL group was shorter than that of the Ho: YAG group [-5.47, 95% CI (-8.86, -2.08), *P* = 0.002]. However, the results demonstrated that the laser time, hospital stay, drop in hemoglobin level, total energy, and postoperative ureteral stenting were similar between the two groups (Table 2).

**Table 2:** Summarized mean differences for perioperative outcomes between TFL and Ho: YAG

	No. of study	heterogeneity	MD	95% CI	*P* value
Operation time	11	*P* < 0.00001, I^2^ = 87%	-5.47	[-8.86, -2.08]	***P* = 0.002**
Laser time^a^	8	*P* < 0.00001, I^2^ = 98%	-0.74	[-1.56, 0.09]	*P* = 0.08
Duration of hospital stay	4	*P* < 0.00001, I^2^ = 91%	-0.20	[-0.89, 0.49]	*P* = 0.57
Drop of hemoglobin^a^	3	*P* = 0.76, I^2^ = 0%	0.22^a^	[-0.05, 0.49]	*P* = 0.12
Total energy	5	*P* = 0.07, I^2^ = 54%	0.02	[-0.26, 0.31]	*P* = 0.88
Postoperative ureteral stenting rate	3	*P* = 0.18, I^2^ = 42%	0.96	[0.84, 1.09]	*P* = 0.50

^a^The two groups used different indicators in the laser time and the drop of hemoglobin, SMD was used as the effect value for the analysis

#### Intraoperative complications

Five studies [23, 26, 27, 30, 32] reported eligible data on intraoperative complications. The findings revealed that the total incidence of intraoperative complications in the TFL group was lower than that in the Ho: YAG group by 8.2% vs. 12.7%, but there was no statistical difference [0.58, 95% CI (0.27, 1.26), *P* = 0.17].

Among the common intraoperative complications, the incidence of stone migration in the TFL group was significantly lower than that in the Ho: YAG group, although the incidence of hematuria, ureteral perforation, and mucosal injury was comparable (Table 3).

**Table 3:** Summarized risk ratios for intraoperative complications between TFL and Ho: YAG

Intraoperative complication	No. of study	heterogeneity	RR	95% CI	*P* value
Hematuria	5	*P* = 0.02, I^2^ = 66%	0.37	[0.12, 1.18]	*P* = 0.09
Ureteral perforation	3	*P* = 1.00, I^2^ = 0%	0.31	[0.05, 1.96]	*P* = 0.21
Mucosal injury	4	*P* = 0.13, I^2^ = 47%	0.85	[0.37, 1.97]	*P* = 0.71
Stone migration	2	*P* = 0.07, I^2^ = 69%	0.17	[0.06, 0.50]	***P* = 0.001**
Total	5	*P* = 0.005, I^2^ = 73%	0.58	[0.27, 1.26]	*P* = 0.17

### Postoperative complications

Eight studies [22, 23, 26–30, 32] examined the overall incidence of postoperative complications. The results showed that there was no statistically significant difference between the two groups in the incidence of postoperative complications [0.95, 95% CI (0.58, 1.55), *P* = 0.84].

The results of the Clavien system for complication evaluation showed that the incidences were similar to Clavien grade 1–2 adverse events, and Clavien grade ≥ 3 adverse events (Table 4).

**Table 4:** Summarized risk ratios for postoperative complications between TFL and Ho: YAG

Postoperative complication	No. of study	heterogeneity	RR	95% CI	*P* value
Total	8	*P* = 0.02, I^2^ = 58%	0.95	[0.58, 1.55]	*P* = 0.84
Clavien grade 1–2	5	*P* = 0.70, I^2^ = 0%	0.88	[0.55, 1.41]	*P* = 0.59
Clavien grade ≥ 3	5	*P* = 0.53, I^2^ = 0%	0.56	[0.19, 1.64]	*P* = 0.29

### Subgroup analysis

The results of subgroup analyses based on stone size, stone location, ureteroscope, study design, follow-up time, MOSES technique, dusting technique, stone density and laser setting are presented in Table 5.

**Table 5:** Subgroup analyses based on stone size, stone location, ureteroscope, study design, follow-up time, MOSES technique, dusting technique, stone density and laser setting for outcomes between TFL and Ho: YAG

Subgroup analysis		SFR	Operation time	Laser time	Intraoperative complications	Postoperative complications
Stone size	stones with diameter > 1 and < 2 cm	1.25 [0.37, 4.21]; *P* = 0.72	-10.69 [-31.43, 10.04]; *P* = 0.31	**-0.86 [-1.42, -0.30]** ***P* = 0.003**	-	-
Stone location	ureter	**0.13 [0.02, 0.75];** ***P* = 0.02**	**-6.52 [-11.85, -1.19];** ***P* = 0.02**	-1.90 [-4.55, 0.75]; *P* = 0.16	**0.42 [0.24, 0.73]** ***P* = 0.002**	**0.43 [0.23, 0.82]** ***P* = 0.01**
	kidney	**0.35 [0.27, 0.46];** ***P* < 0.0001**	-1.31 [-4.87, 2.26]; *P* = 0.47	0.25 [0.09, 0.40]; *P* = 0.002	2.10 [1.01, 4.38]; *P* = 0.05	1.85 [1.28, 2.68]; *P* = 0.001
	ureter/kidney	0.70 [0.35, 1.41]; *P* = 0.32	**-7.06 [-13.70, -0.42];** ***P* = 0.04**	-0.22 [-0.71, 0.27]; *P* = 0.38	**0.42 [0.22, 0.78];** ***P* = 0.007**	1.06 [0.62, 1.81]; *P* = 0.82
Ureteroscope	semi-rigid ureteroscope	**0.10 [0.01, 0.77];** ***P* = 0.03**	-3.30 [-8.73, 2.12]; *P* = 0.23	-1.90 [-4.55, 0.75] *P* = 0.16	**0.45 [0.25, 0.80];** ***P* = 0.007**	0.52 [0.26, 1.04]; *P* = 0.06
	rigid ureteroscope	0.27 [0.01, 6.59]; *P* = 0.43	**-17.70 [-22.55, -12.85];** ***P* < 0.00001**	-	0.21 [0.02, 1.77] *P* = 0.15	0.14 [0.02, 1.10] *P* = 0.06
	flexible ureteroscope	**0.35 [0.27, 0.46];** ***P* < 0.00001**	-5.07 [-13.42, 3.28]; *P* = 0.23	-0.11 [-0.73, 0.50]; *P* = 0.72	2.10 [1.01, 4.38]; *P* = 0.05	1.85 [1.28, 2.68]; *P* = 0.001
Study design	RCT	0.51 [0.16, 1.61]; *P* = 0.25	**-5.82 [-10.60, -1.04]** ***P* = 0.02**	-1.23 [-2.76, 0.30] *P* = 0.12	**0.40 [0.24, 0.66];** ***P* = 0.0003**	0.70 [0.43, 1.14]; *P* = 0.15
	non-RCT	**0.48 [0.28, 0.81];** ***P* = 0.006**	-5.24 [-11.26, 0.78]; *P* = 0.09	0.05 [-0.31, 0.42]; *P* = 0.78	0.79 [0.23, 2.70] *P* = 0.71	1.21 [0.62, 2.37]; *P* = 0.57
Follow-up time	< 3 months	0.69 [0.29, 1.62]; *P* = 0.39	-3.52 [-9.31, 2.27]; *P* = 0.23	-1.29 [-3.34, 0.76]; *P* = 0.22	**0.45 [0.25, 0.80];** ***P* = 0.007**	0.64 [0.36, 1.14]; *P* = 0.13
	≥ 3 month	**0.41 [0.22, 0.77];** ***P* = 0.005**	-4.06 [-9.26, 1.13]; *P* = 0.13	-0.14 [-0.67, 0.39]; *P* = 0.61	0.61 [0.21, 1.75]; *P* = 0.36	1.16 [0.66, 2.05]; *P* = 0.60
MOSES technique	with MOSES	0.70 [0.10, 4.76]; *P* = 0.72	-2.08 [-6.38, 2.22]; *P* = 0.34	0.07 [-0.31, 0.45]; *P* = 0.71	-	1.15 [0.59, 2.22]; *P* = 0.68
	without MOSES	**0.47 [0.29, 0.76];** ***P* = 0.002**	**-5.90 [-9.58, -2.22];** ***P* = 0.002**	-0.86 [-1.82, 0.10]; *P* = 0.08	0.58 [0.27, 1.26]; *P* = 0.17	0.84 [0.43, 1.64]; *P* = 0.61
Dusting technique	with dusting	**0.34 [0.26, 0.44];** ***P* < 0.00001**	-2.55 [-5.77, 0.67]; *P* = 0.12	0.21 [0.06, 0.35]; *P* = 0.005	0.83 [0.13, 5.36]; *P* = 0.84	1.68 [1.19, 2.36]; *P* = 0.003
	without dusting	0.85 [0.59, 1.22]; *P* = 0.38	**-7.19 [-7.81, -6.56];** ***P* < 0.00001**	**-0.76 [-0.96, -0.56];** ***P* < 0.00001**	**0.45 [0.28, 0.73];** ***P* = 0.001**	0.69 [0.45, 1.07]; *P* = 0.10
Stone density	< 1000Hu	**0.25 [0.10, 0.63]** ***P* = 0.003**	**-8.00 [-15.46, -0.54]** ***P* = 0.04**	0.00 [-0.36, 0.36] *P* = 1.00	**0.31 [0.12, 0.80]** ***P* = 0.02**	0.88 [0.34, 2.26] *P* = 0.78
	≥ 1000Hu	**0.57 [0.33, 0.97]** ***P* = 0.04**	**-5.27 [-8.88, -1.66]** ***P* = 0.004**	-0.85 [-1.82, 0.12] *P* = 0.09	0.68 [0.28, 1.68] *P* = 0.40	0.94 [0.54, 1.65] *P* = 0.84
Laser setting	low energies, high frequencies	**0.50 [0.30, 0.83]** ***P* = 0.008**	-1.79 [-5.37, 1.79] *P* = 0.33	0.15 [-0.35, 0.65] *P* = 0.56	-	1.17 [0.52, 2.64] *P* = 0.71
	high energies, low frequencies	0.36 [0.10, 1.35] *P* = 0.13	**-6.73 [-11.47, -1.99]** ***P* = 0.005**	-1.11 [-2.53, 0.31] *P* = 0.13	**0.38 [0.24, 0.62]** ***P* < 0.0001**	**0.61 [0.38, 0.97]** ***P* = 0.04**

## Discussion

### Main findings

Since the late 1990s [34, 35], when laser lithotripsy was introduced into urology practice, the Ho: YAG has been the gold standard treatment for endoscopic lithotripsy. The TFL has gained increased attention for its role in stone lithotripsy since its first reported application in stone lithotripsy in 2005 [36]. Since then, an increasing number of studies have investigated its use in preclinical and clinical settings.

This systematic review included 13 studies with 1394 patients. The efficacy and safety of TFL and Ho: YAG in treating urinary stones were compared. The main finding of this systematic review was that TFL was associated with a significantly higher SFR, shorter operation time, and less stone migration than Ho: YAG. However, TFL had no benefits related to the laser time, duration of hospital stay, drop in hemoglobin level, total energy, postoperative ureteral stenting, incidence of intraoperative complications or postoperative complications.

### SFR

The primary goal of all stone removal procedures is to achieve the highest stone clearance rate (SFR). In our study, TFL was found to be associated with a significant improvement in SFR. This might be related to better lithotripsy efficiency, smaller stone fragments, and finer stone powder. Laboratory studies have shown that the TFL produces smaller stone fragments than Ho: YAG, which is more conducive to fragment clearance [37–39]. Jones's review also confirms this conclusion [2]. Besides, TFL can be delivered through thinner fiber, which improves irrigation and visibility without loss of flexibility, even if the fiber is thin [40]. In addition, the SFR may be related to various factors, including the surgeon's experience, stone size, stone location, and stone density [2].

### Perioperative outcomes

In our study, the operation time were shorter in the TFL group. This could be due to the technical advantages of TFL proven in the laboratory, such as higher ablation speed, higher ablation efficiency, and less stone retropulsion, which can considerably reduce the time required for operation [41, 42]. In addition, TFL could pulverize the stones without the need to use a stone basket to remove stone fragments, which greatly reduce the operation time.

We found no significant differences in the laser time, duration of hospital stay, drop in hemoglobin level, total energy, or postoperative ureteral stenting. This might be because both the TFL and Ho: YAG groups belong to laser lithotripsy technology, and the current diagnosis and treatment process of laser lithotripsy is relatively standard. These results did not change and remained similar to the overall results even after multiple subgroup analyses were performed. Among all the included studies, all patients in Christopher’s [29] study had day care surgery, and we did not include the data of this study in the analysis of hospital stay. However, it has been shown that URSL can be used as a day surgery as long as the procedure is successful and without complications [43, 44]. Perhaps to standardize the surgical process, Ankit and Bogdan [22, 28] placed stents after surgery in all the patients who participated in the experiment. We did not include the data of the two studies in the analysis of postoperative ureteral stenting.

### Intraoperative complications

Among intraoperative complications, the incidence of stone migration was significantly reduced in the TFL group. This result was predictable, which was confirmed by showing that TFL was related to less stone retropulsion in vitro experiments [41, 42]. Preclinical studies have shown the shorter water-borne optical penetration depth of TFL, resulting in a higher damage threshold for peripheral tissue [7]. In addition, TFL produced finer stone powder, which prevented repeated application of the stone basket and reduced the risk of ureteral injury [3]. However, our study showed that TFL had a lower overall rate of intraoperative complications but was not statistically significant, and there was no significant difference in hematuria, ureteral perforation, and mucosal injury between the two groups. A significant difference was not reached due to the small number of studies included although there might be advantages of TFL. However, due to the lack of studies reporting other complications, more trials and standardization of postoperative complications are needed to confirm this idea.

### Postoperative complications

No significant differences in postoperative complications were found between the two groups, even after grading with the Clavien system. Laser lithotripsy is a highly effective and safe technique for the treatment of urinary stones in virtually any patient population. Except in rare cases, the occurrence of postoperative complications might be dependent on various other factors associated with the procedure, including the surgeon's experience, stone size, location, and density [2, 45].

### Subgroup analysis

#### Stone location

Our results showed that TFL led to a higher SFR, lower intraoperative and postoperative complications when treating patients with ureteral stones alone. This might be related to the fact that TFL could promote higher lithotripsy efficiency, lower tissue injury, thinner fiber, and better surgical vision [40, 45, 46].

#### Stone density

The American Urological Association guidelines suggested that RIRS was more appropriate for kidney stones with densities greater than 1000 HU to avoid additional surgeries [47, 48]. Therefore, we used a critical value of 1000 HU for stone density subgroup analysis. Our results showed that the advantages in terms of SFR and operative time for TFL did not change in the variation of stone density. In addition, lower intraoperative complications were found when the stone density was lower.

#### Ureteroscope type

SFR had advantages in the applied semi-rigid and flexible ureteroscope subgroups, but there was no difference in the rigid ureteroscope group. This might be related to the fact that the semi-rigid and flexible ureteroscope are mostly used in the middle and upper ureteral stones or difficult stones such as the stones moving up to the kidney. The larger diameter of the rigid ureteroscope was mostly used for lower ureteral stones. As we all know, laser fiber also played a vital role in lithotripsy. The thicker laser fibers might impede the flow of irrigation fluid, limit the flexibility of the instrument, and reduce the surgical vision [37, 49]. The thinner TFL fiber facilitated flexible operation, especially in flexible ureteroscopy. These results suggested that TFL might be more appropriate for patients with complex stones such as upper stones and mixed stones.

#### Laser setting

Laser lithotripsy with higher energies (> 0.5 J) speeded up the process of breaking up large stones into smaller pieces. The combination of low energy (0.2–0.5 J) and high frequency (40–50 Hz) caused the stone to be crushed into fine particles [45]. We set 0.2–0.5 J and 40–50 Hz as thresholds for low energy and high frequency for subgroup analysis of laser settings. Our results showed that TFL in low energy and high frequency had advantages in SFR. In contrast, TFL in high energies had the advantages in terms of operative time, intraoperative complications and postoperative complications. This was acceptable because laboratory results showed that TFL was related to less tissue injury than Ho: YAG at the same high energy [7].

#### MOSES technique

MOSES technique is a new pulse modulation technique. Our results showed that TFL had advantages in terms of SFR and operative time compared with Ho: YAG without Moses technique, but these advantages disappeared when compared with Ho: YAG with Moses technique. The in vitro studies had shown that Ho: YAG with Moses had higher stone ablation rate and less stone retropulsion than traditional Ho: YAG [50, 51].

#### Dusting technique

Our results showed that TFL had advantage for SFR in applied dusting technique, but no difference in without dusting technique. Dusting technique promoted smaller stone fragments so they could be naturally eliminated, which reduced the use of stone baskets and associated complications [45].

### Results in relation to other studies and reviews

The relevant studies by Traxer et al. [39], Jones et al. [2] and Chua et al. [52] are all reviews of the latest applications of TFL. Traxer et al. reported that TFL shortened the operation time, which was consistent with our findings. Jones et al. suggested that TFL appears to be effective in stone lithotripsy. However, their studies comprised few clinical studies, with the majority being comprehensive analyses of previously available laboratory or in vitro experiments. The study by Traxer et al. [39] consisted of seven full-text clinical trials and 18 laboratory studies. The study by Jones et al. [2] consisted of 11 clinical studies. Furthermore, most of the included clinical studies were case reports or only reported the application of TFL without comparison with Ho: YAG. In the study by Chua et al. [52], 15 articles were included, two of which used TFL for stone treatment in PCNL. It was a completely different surgical approach from URS, which would have a significant impact on SFR, operation time, and other results. Therefore, our systematic review, which integrated all available clinical evidence, is particularly significant.

### Strengths and limitations

Compared to prior meta-analyses, this systematic review has several advantages. First, clinical studies that focused on comparing TFL and Ho: YAG in the treatment of urolithiasis were analyzed, which was not previously available. The previous meta-analysis focused on the application of TFL but did not compare them with Ho: YAG. We considered more results than previous studies, which can provide a more comprehensive view of efficacy and safety. Second, our search strategy was rigorously designed to find more eligible clinical studies. Third, considering the large population data in China, we searched several Chinese databases and included Chinese articles in our systematic review. Fourth, we performed subgroup analyses based on various variables to synthesize the risk of bias and assess the strength of the available evidence.

Our study has certain limitations. First, there was considerable heterogeneity among the included studies. This could be attributed to differences in study design, population, region, equipment used, and surgeon experience, which might have influenced the results. Second, the overall sample size was still small. The sample size of some studies, such as those associated with stone migration and mucosal injury, may have influenced our results. Third, there were a small number of included studies with a follow-up time of fewer than three months.

### Implications for research and practice

Our systematic review might also have implications for further research and clinical practice. Future studies should focus on large, high-quality randomized controlled trials of stone location, stone size, laser setting and type of ureteroscopy to clarify the efficacy, safety, potential advantages and disadvantages and to evaluate the impact of long-term and related complications of TFL compared with Ho: YAG.

Although the traditional Ho: YAG is still considered the “gold standard” in clinical guidelines, our results demonstrate that TFL might be a more effective and safer in URS lithotripsy for low-density stones or high-energy laser setting, especially for middle and upper ureteral stones. In addition, our results show that SFR is improved in dusting mode. TFL has advantages in stone clearance rate, operative time, intraoperative complications and postoperative complications in ureteral stones. These results suggest that TFL may be more suitable for patients with ureteral stones.

## Conclusions

In summary, our study provides strong available evidence that TFL provides higher SFR, shorter operative time and less stone migration suggesting that TFL may be more effective and safer than Ho: YAG. With further development, TFL may become a more effective alternative to traditional Ho: YAG for laser lithotripsy.

## Data Availability

The datasets used and/or analyzed during the current study are available from the corresponding author on reasonable request.

## Abbreviations

TFL: Thulium fiber laser
Ho: YAG: Holmium: YAG
SFR: Stone-free rate
RCT: Randomized controlled trial
CENTRAL: The Cochrane Central Register of Controlled Trials
CNKI: Chinese National Knowledge Infrastructure
NOS: Newcastle‒Ottawa Scale
RR: Relative risks (s)
WMD: Weighted mean difference
CI: 95% Confidence intervals
SD: Standard deviation
NA: Not available
NR: Not reported
